# Is it sufficient to evaluate metastatic bone involvement in breast cancer using SPECT/CT? A new approach of SPECT/CT-guided targeted bone marrow biopsy

**DOI:** 10.1186/s12885-022-09702-1

**Published:** 2022-06-04

**Authors:** Xiaomin Li, Caixia An, Wanchun Zhang

**Affiliations:** 1 Shanxi Bethune Hospital, Shanxi Academy of Medical Sciences, Tongji Shanxi Hospital, Third Hospital of Shanxi Medical University, Longcheng Street NO.99, Taiyuan, 030032 China; 2 Tongji Hospital, Tongji Medical College, Huazhong University of Science and Technology, Wuhan, 430030 China

**Keywords:** Bone metastasis, Breast cancer, Biopsy, Tomography, Mission computer, Single photon, 99Tcm-methyl diphosphonate

## Abstract

**Objective:**

To investigate the feasibility, safety, and clinical application value of single photon emission computed tomography/computed tomography (SPECT/CT)-guided bone marrow biopsy (BMB) in breast cancer (BC) patients with suspected bone metastases (BM) and compare its diagnostic performance for detection of BM with SPECT/CT.

**Methods:**

The records of breast cancer patients referred for bone scintigraphy (BS), SPECT/CT and SPECT/CT-guided BMB from January of 2018 to June of 2021 in our hospital were retrospectively reviewed. 49 Patients were consecutively included in this study, all 49 specimens were analyzed by pathological and immunohistochemical studies.The biopsy success rate, total examination time, biopsy operation time, complications, CT radiation dose, and pathological and immunohistochemical results were recorded. The diagnostic performance based on SPECT/CT and SPECT/CT-guided BMB were compared with pathological, immunohistochemical examinations and the results of subsequent follow-up.

**Results:**

Bone samples of the sites with high uptake were obtained in all 49 patients under BMB. No severe postoperative complications occurred. Among all 49 cases, 34 specimens were positive for metastatic breast cancer (69%, 34/49), and positive for benign tissue in 15 cases (31%, 15/49). 1 case of 15 cases was subsequently diagnosed as metastatic breast cancer according to the follow-up result. SPECT/CT-guided BMB demonstrated significantly higher negative predictive value (NPV) when compared to SPECT/CT (*p* = 0.021 < 0.05). Patients with differential expression of ER, PR, and HER-2 between primary lesions and metastatic lesions accounted for 12, 17, and 5 cases, respectively, and the changing rates were 35.2% (12/34), 50% (17/34), and 14.7% (5/34), respectively. Molecular subtype changes occurred in 7 patients, accounting for 47% (16/34) of metastatic patients.

**Conclusion:**

It is insufficient to evaluate BM in BC patients using SPECT/CT imaging. SPECT/CT-guided BMB provided significantly higher sensitivity and NPV than SPECT/CT for detection of BM in BC patients. Our research redefines a new approach which can confirm diagnosis and potential molecular subtype changes for suspected bone metastatic lesions in BC patients, which can offer important opportunities for precision treatment and improved quality of life of BC patients with BM.

## Background

Breast cancer (BC) is the most common malignant tumour among women [1]. Bone is the most common location of metastases for BC. Bone metastases (BM) count approximately 60–70% of all metastatic BC and more than 70% of patients showed bone metastases during autopsy [2]. BM significantly affects both quality of life and survival of the breast cancer patient. Clinically, complications secondary to BM include pain, pathologic fractures, spinal cord compression, and hypercalcemia of malignancy [3]. Therefore, early diagnosis and treatment of BM in breast cancer patients has important significance.

Bone marrow biopsy (BMB) is the “gold standard” of diagnosis of BM in BC. According to relevant practice guidelines of the National Comprehensive Cancer Network (NCCN), European Society for Medical Oncology (ESMO)and China Anti-cancer Association (CACA) [4, 5], re-biopsy for suspected metastatic lesions in patients with late-stage BC is considered to confirm diagnosis. The evaluation of BM status is of critical importance in BC, as it is re-evaluated to confirm potential molecular subtype changes. The results may directly change the treatment plan.

However, It is not always performed in routine practice due to expertise unavailable and the lack of special technologie. In addition to the difficulties performing a re-biopsy in a location that is difficult to access, conventional imaging such as CT may not identify a metastasis and thus a biopsy will not be performed as a routine practice [6].

Nuclear medicine molecular imaging has unique advantages with respect to target area selection. In previous study, PET/CT-guided targeted BMB was confirmed to be a safe and feasible technique for the appraisal of advanced lung cancer and lymphomas [6, 7]. Zhao et al. applied SPECT/CT for thoracic tumor biopsy and confirmed its safety and reliability [8]. However, there are no studies examining the utility of SPECT/CT-guided targeted BMB.

After mastering PET/CT-guided percutaneous biopsy technology, this technology was introduced into SPECT/CT to perform SPECT/CT-guided BMB to target suspected bone metastatic lesions in breast cancer and test its feasibility and clinical value. The results are reported below.

## Methods

### Patients

The records of women with biopsy-proven breast cancer referred for routine clinical work-up with 99Tcm-methyl diphosphonate (MDP) bone scintigraphy (BS) and SPECT/CT from January of 2018 to June of 2021 in our hospital were retrospectively reviewed. Patients were consecutively included in this retrospective study if positive lesions were identified on SPECT/CT imaging and SPECT/CT-guided BMB were performed, and patients with a second malignancy were excluded. SPECT/CT scans were performed within 7 days before BMB. The findings from SPECT/CT and SPECT/CT-guided BMB were compared with the results of subsequent imaging follow-up and pathological and immunohistochemical examinations.

### 99mTc-MDP SPECT/CT technique and imaging

A GE Discovery NM/CT670 combined with a low energy high resolution collimator, with an energy window of 20% and energy peak of 140 keV, was used. 99mTc-MDP 740–1110 MBq was intravenously injected, and anterior and posterior full-body images and SPECT/CT fusion images were collected after 3 h. After determining the location of scanning field, SPECT tomography was performed first, with a matrix of 128 × 128, continuous acquisition of 360°, rotation of two probes of 180°, 12 s for each frame, a total of 32 frames were collected. Then, CT scanning was performed automatically with a matrix of 256 × 256 and scanning layer thickness of 5 mm(CT scanning parameters: 120 kV, 80 m A, 25 mm/ s entering speed, layer thickness 3.75 mm). Volumetrix MI Evolution for Bone was used for image reconstruction and fusion, without attenuation correction, Butterworth filter function, cutoff frequency 6.0, and transverse, sagittal, coronal and 3D images of SPECT, CT and their fusion were obtained directly. The matrix, pixel size and effective frame number of reconstructed SPECT and CT images are identical.

### Image analysis

BS and SPECT-CT images were independently analyzed by two experienced nuclear medicine physicians on the work station. The readers were blinded to patients’ clinical information including previous therapy, previous BS findings, and the findings of other imaging modalities. Only the lesions that were not clearly defined on BS were evaluated. In case of any discrepancy regarding the findings of planar and SPECT images, a consensus was reached after mutual discussion. Malignant lesions were suggested by the presence of lytic, sclerotic, or mixed lytic-sclerotic changes on CT images. The presence of osteophytes, spondyloarthropathy, subchondral sclerosis, or narrowing of the joint space was regarded as a clear sign of the benign nature of the lesion. We also identified the location and pattern of bone lesions that was recorded.

### SPECT/CT-guided targeted BMB

99mTc-MDP SPECT/CT fused images were used to determine the appropriate puncture site with high uptake of MDP, and the biopsy needle was introduced stepwise under fused SPECT/CT image and CT guidance. After conventional disinfection, draping, and local anaesthesia to the periosteum using 1% lidocaine, a bone puncture needle (BMT-B 2.4 × 70, Shanghai SA Medical Technology) was pressed, rotated, and inserted in accordance with the plan. Scanning was performed again to confirm that the needle tip was located at the edge of the target area (120 kV, 20 mA, image fusion using VMI software). The needle was connected to a spare casing tube and inserted into the needle core. The needle was forcefully pressed and rotated clockwise. When the fusion image confirmed that the needle tip passed through the target area, tissues were obtained after the needle was rotated counterclockwise and withdrawn. One or two samples were obtained for each patient, and the lengths of samples all were 1.5 or 2.2 cm. The BMB specimens were fixed in 10% formaldehyde solution and analyzed by morphological and immunohistochemical studies. The pathological results of all BMBs were validated by review of the individual pathology reports. After the biopsy procedure was finished, patients were kept for observation for at least 30 min after the in a recovery room and were allowed to leave when there were no adverse reactions.

### Molecular subtyping of groups

Based on estrogen receptor (ER), progesterone receptor (PR) and human epidermal growth factor receptor 2(HER2) status, patients were classified following the recommendations of the 12th International Breast Conference [9]. The five patient groups were: 1) Luminal A: ER( +) and/or PR( +), HER2(-),Ki-67 low (< 14%); 2) Luminal B-HER2(-): ER( +) and/or PR( +),HER2(-), and Ki-67 high (> 14%); 3) Luminal B-HER2( +): ER( +)and/or PR( +), HER2( +), and any Ki-67 index; 4) HER2( +): ER(-),PR(-), and HER2( +); 5) Basal: ER(-), PR(-), and HER2(-).

### Follow-up and reference standard

All the previous clinicopathological data of 49 patients have been followed up as soon as possible and their molecular subtypes have been classified. We derived the final diagnoses from histopathology and clinical/imaging follow-up (CT, MRI, PET -CT, SPECT -CT) over at least 6 months. It was considered positive for a tumor if there is an increase in size or a change of nature under treatment, whereas benign if lesions had unchanged size and character over 6 months without therapy [10].

### Statistical analysis and ethics

The total examination time, biopsy operation time, complications, CT radiation dose, biopsy success rate and the changing rate were recorded. The changing rate was equal to the number of patients with altered expression of ER, PR, HER2 or molecular subtype divided by the total number of patients undergoing immunohistochemistry. The total dose-length product (DLP; mGy) of each scan was used as the CT radiation dose that patients received. The effective radiation dose (DLP × weighting factor κ; 0.019 mSv•mGy-1•cm-1 for the chest and 0.016 mSv•mGy-1•cm-1 for the abdomen and pelvis) was calculated. According to the the final diagnoses from histopathology and clinical/imaging follow-up, the diagnosis of SPECT/CT and SPECT/CT-guided BMB were classified as true-positive (TP), false-positive (FP), true-negative (TN), and false-negative (FN). Sensitivity, specificity, positive predictive value (PPV) and negative predictive value (NPV) were is calculated according to the number of TN, TP, FP, FN, and determined on the basis on number of patients, not number of lesions. McNemar test was used to test differences in the sensitivity and specificity between SPECT/CT and SPECT/CT-guided BMB. Chi-square test was used to test differences in the NPV and PPV between SPECT/CT and SPECT/CT-guided BMB. All statistical analysis was performed using SPSS, version 26 software. A *p*-value < 0.05 was considered significant. This retrospective evaluation of collected data was approved by the ethics committee of our institution. This study was approved by the Ethics Committee of our hospital (approval number: YXLL-2020–033).

## Results

### SPECT/CT-guided BMB

Bone samples of the sites with high uptake of MDP were obtained in all 49 patients under BMB. Of these, biopsy tissue was successfully obtained in 10% of patients (5/49) despite the absence of morphological lesions on CT images. The average total examination time was (42.3 ± 10.8) min, the average biopsy operation time was (24.5 ± 6.2) min, and the effective radiation dose was (1.9 ± 0.8) mSv. No postoperative complications such as infection, pneumothorax, massive bleeding, or nerve damage occurred.

The sites and pattern of biopsy lesions are summarized in Table 1.

**Table 1:** Sites and CT pattern of evaluated lesions

Vriable	n	%
Sites	49	100
Vertebrae	13	26
Pelvis	12	24
Scapula	3	6
Sternum	11	22
Ribs	6	12
Clavicle	4	8
Characters		
Lytic	26	53
Sclerotic	18	36
Unchanged	5	11

### Pathological diagnosis of BM

Among all 49 cases, 34 specimens were positive for metastatic breast cancer (69%, 34/49), and positive for benign tissue in 15 cases (31%, 15/49).

Biopsy identified 15 benign bone lesions, including fracture and bone marrow tissues (*n* = 10), fibrous tissues (*n* = 2), inflammatory cell infiltration (*n* = 3), myofibroblastoma (*n* = 1). To avoid potentially false negative of the 15 cases, they were further evaluated by clinical and imaging follow-up: 14 cases were confirmed as benign (inflammatory, myofibroblastoma, and lymph node hyperplasia) and 1 cases were subsequently diagnosed as metastatic breast cancer.

**Fig. 1:**
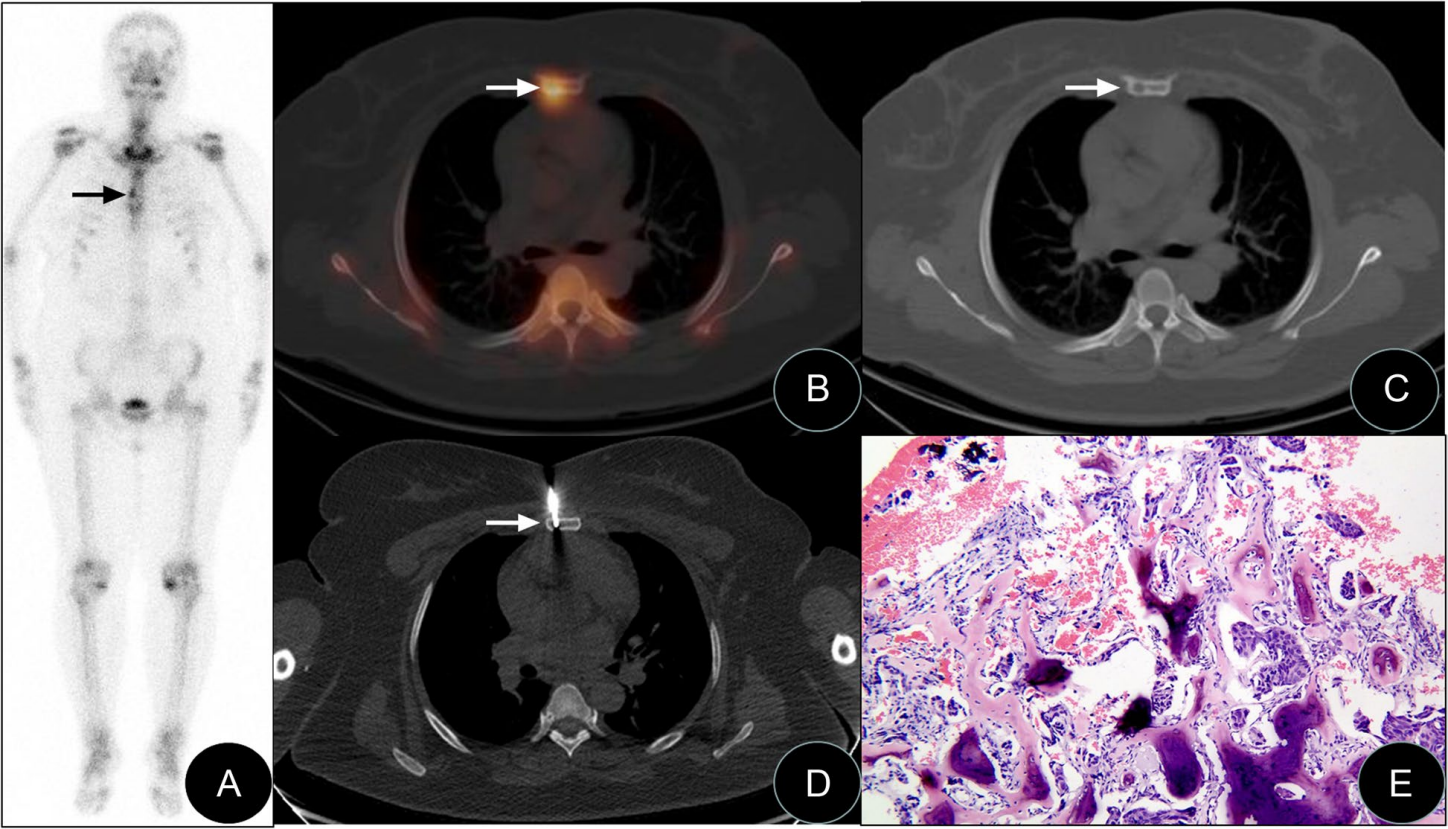
SPECT/CT-guided targeted bone marrow biopsy in the sternal body. Anterior bone scintigraphy, SPECT/CT fusion tomography image, biopsy image, and biopsy pathology image of a patient with invasive ductal carcinoma of the left breast (female, 44 years old). **A** The anterior bone scintigraphy suggested increased focal abnormal metabolism in the sternal body (black arrow). **B**, **C** SPECT/CT fusion tomography showed bone destruction in a location of increased metabolism in the sternal body. **D** SPECT/CT-guided percutaneous biopsy of the biological target in the sternal body. **E** Pathological examination results suggested metastatic breast invasive ductal carcinoma, and light microscopy results revealed the nested distribution of tumour cells in the space between bones and dead bones, with obvious cell atypia combined with nuclear hyperchromic malformation (HE × 100)

**Fig. 2:**
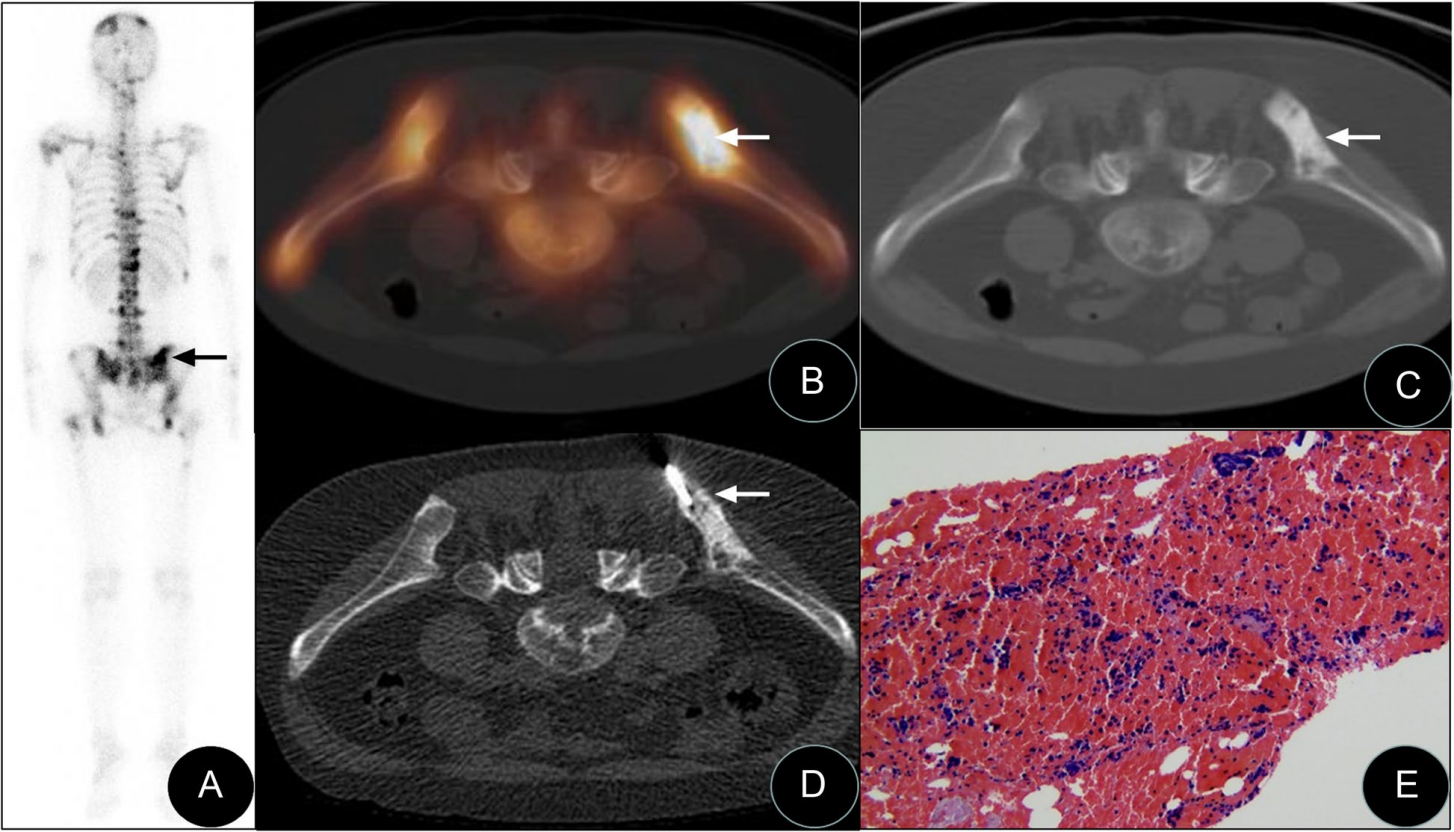
SPECT/CT-guided targeted bone marrow biopsy in the right ilium. Posterior bone scintigraphy, SPECT/CT fusion tomography image, biopsy image, and biopsy pathology image of a patient with invasive ductal carcinoma of the right breast 3 years after surgery (female, 29-year-old). **A**. The posterior bone scintigraphy suggested multiple abnormal increases in bone metabolism in the whole body and an abnormal increase in metabolism of the right ilium (black arrow). **B**, **C** SPECT/CT fusion tomography results indicated that the bone density in the right ilium that exhibited increased metabolism had significantly increased. **D** SPECT/CT-guided percutaneous biopsy of the biological target in the right ilium. **E** Pathological examination results suggested metastatic breast invasive ductal carcinoma, and light microscopy results revealed metastatic adenocarcinoma cells in blood clots (HE × 100)

**Fig. 3:**
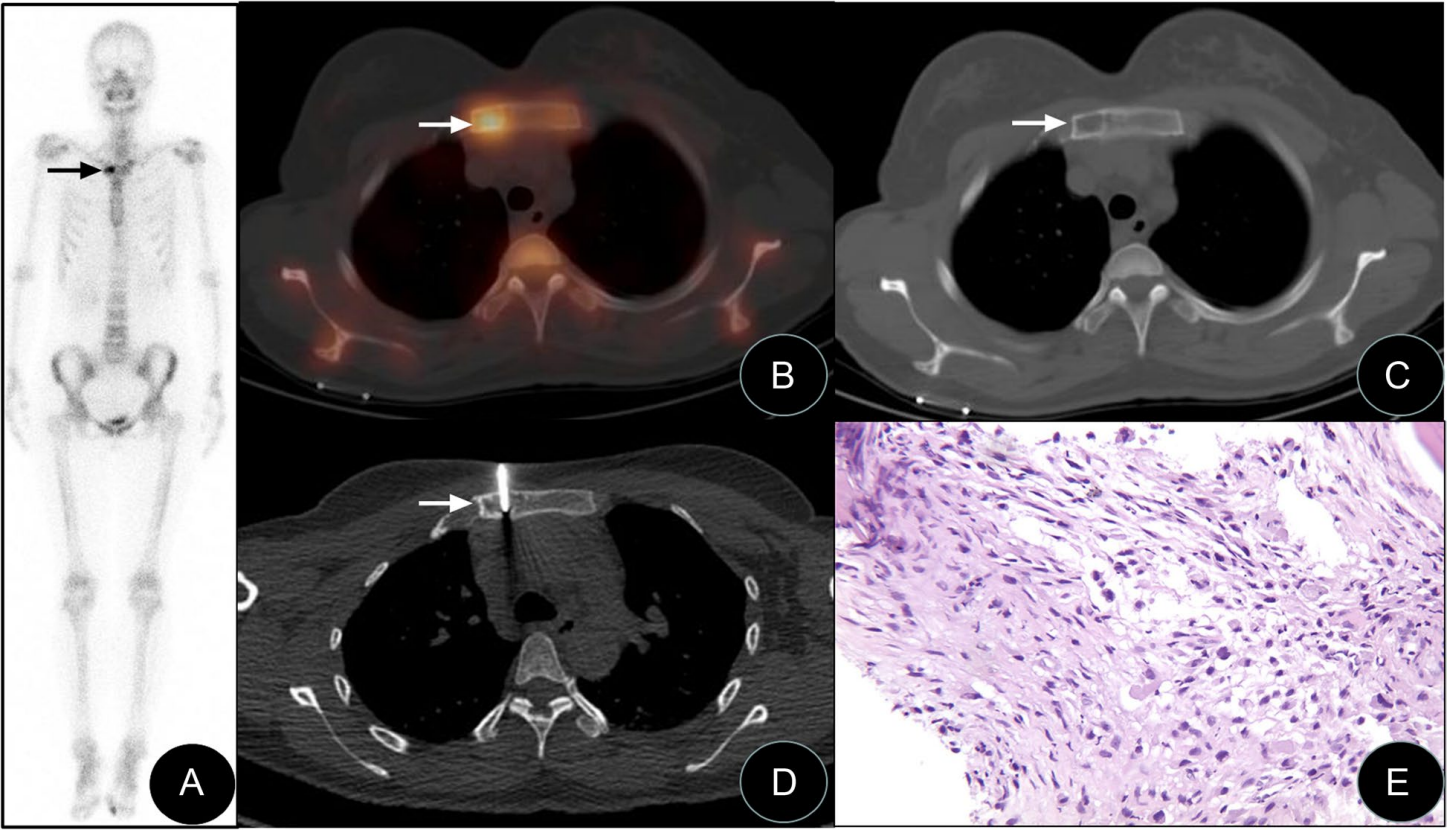
SPECT/CT-guided targeted bone marrow biopsy in the manubrium. Anterior bone scintigraphy, SPECT/CT fusion tomography image, biopsy image, and biopsy pathology image of a patient with invasive ductal carcinoma of the right breast 2 years after surgery (female, 30-year-old). **A** The anterior bone scintigraphy suggested abnormally increased bone metabolism on the right side of the manubrium (black arrow). **B**, **C** SPECT/CT fusion tomography results showed bone destruction in the location of increased metabolism on the right side of the sternal body. **D** SPECT/CT-guided percutaneous biopsy of the biological target in the sternal body. **E** Pathology and immunohistochemistry results suggested myofibroblastoma. Light microscopy results revealed a small amount of bone and dead bone tissues, polygonal cells between bone trabeculae, and nucleoli (HE × 100)

Typical images are shown in Figs. 1, 2 and 3.

### Diagnostic performance of SPECT/CT vs. SPECT/CT-guided BMB

According to the pathological results and follow-up results, BM were confirmed in 35 of the 49 patients (71%). SPECT/CT was positive for disease in 27, yielding a sensitivity of 77% (27/35). Because of 1 case was false negative, the sensitivity of SPECT/CT-guided BMB was 97.5% (34/35). SPECT/CT-guided BMB exhibited significantly higher sensitivity when compared to SPECT/CT for determination of BM (*p* = 0.016 < 0.05) (Table 2).

**Table 2:** Sensitivity, specificity, positive predictive value, negative predictive value, and accuracy of SPECT/CT, and SPECT/CT-guided BMB in diagnosing metastasis

Parameter	SPECT/CT	SPECT/CT-guided BMB	*P* value
Sensitivity	77	97.5	0.016*
Specificity	71	100	
PPV	87	100	
NPV	55.5	93.3	0.021*
Accuracy	79	98	

*CT* Computed Tomography, *NPV* Negative Predictive Value, *PPV* Positive Predictive Value, *SPECT* Single-photon Emission Computed Tomography

SPECT/CT was true negative for BM in 10 of 14 patients, yielding a specificity of 71% (10/14), whereas the specificity was of SPECT/CT-guided BMB (14/14,100%). (Table 2).The PPV and NPV of SPECT/CT were 87% (27/31) and 55.5% (10/18) respectively, while PPV and NPV of SPECT/CT-guided BMB were 100% (34/34) and 93.3% (14/15) respectively. SPECT/CT-guided BMB demonstrated significantly higher NPV when compared to SPECT/CT (*p* = 0.021 < 0.05) (Table 2).

### Molecular classification & molecular subtyping shifts

Immunohistochemical examinations of ER, PR, and HER-2 were performed for all 34 metastatic tumors. Changes in ER expression in metastatic lesions and primary lesions were discovered in 12 patients (7 patients converted from positive to negative, and 5 patients converted from negative to positive), and the changing rate was 35.2%. Shifts in PR expression were discovered in 17 patients (13 patients converted from positive to negative, and 4 patient converted from negative into positive), and the changing rate was 50%. Conversions in HER-2 expression were discovered in 5 patients (4 patients converted from positive to negative, and 1 patient converted from negative to positive), and the changing rate was 14.7%. According to diverse combinations of ER, PR and HER-2 status, the total molecular subtype rate of molecular subtype shifts was up to 47% (16/35) (Table 3).

**Table 3:** Molecular subtyping shifts of 34 breast cancer patients

Molecular subtype shifts	datum	%
Unchanged	19	55.8
Changed	16	47.0
Total	34	100

## Discussion

To our knowledge, this study is the first to propose the notion that SPECT/CT-guided targeted BMB, which can add pathological confirmation and monitor potential molecular subtyping shifts if positive bone metastatic lesions were identified on SPECT/CT imaging. Based on the above data that we achieved, SPECT/CT-guided targeted BMB has gradually become routine practice at our institution.

Breast cancer is an evolutionary heterogeneous tumour, and its molecular subtype can convert between bone metastatic lesions and primary tumors [11, 12]. Therefore, it is significant that early detection of BM and personalized treatment based on molecular subtypes, which can preserve or improve long-term quality of life and functional independence of BC patients with BM [13].

Relevant practice guidelines of the National Comprehensive Cancer Network (NCCN), European Society for Medical Oncology (ESMO) and China Anti-cancer Association (CACA) all recommend re-biopsy for suspected metastatic lesions in patients with late-stage breast cancer to confirm diagnosis. After metastasis is confirmed, biological indicators are re-evaluated to confirm potential molecular subtype changes. The results may directly change the treatment plan.

CT guided-BMB is currently the main guidance method for bone biopsy. J. F. Hilton assessed the samples of patients underwent CT-guided biopsy of a radiologically evident BM from breast cancer. Positive samples for metastatic breast cancer were 21/39(52.5%) and sufficient tumor cells for hormone receptor analysis were available in 19/39(48.8%) [14]. However, there are some limitations [15]: (1) CT cannot confirm the sampling target for tumors that do not have lesions with abnormal morphology and structure at the early stage. (2) Systemic staging information cannot be obtained, the safest target area cannot be selected.

Nuclear medicine molecular imaging, including SPECT/CT and PET/CT, can display anatomic and metabolic information concurrently and has unique advantages with respect to target area selection.

Bone scintigraphy (BS),which is different from anatomical imaging,is a kind of imaging examination based on its own function. Metastatic bone tumors are usually detected 3 to 6 months earlier than CT. BS plays an irreplaceable role in the screening and early diagnosis of BM [16]. SPECT/CT has realized the organic combination of metabolic imaging and anatomical imaging, which has important clinical value in differentiating benign and malignant bone lesions [17, 18]. For staging of the skeleton, because of the greater contrast resolution of SPECT coupled with the correlation with the morphologic appearance of lesions on CT, further gains in sensitivity and, especially, in specificity and diagnostic confidence were apparent with SPECT/CT [10, 19, 20].

Many studies showed that PET/CT for guidance or guiding biopsy is feasible and may optimize the diagnostic yield of image-guided interventions [21, 22]. Wei et al. reported that PET/CT-guided percutaneous FDG-avid target biopsies offers a new integrated precise re-biopsy algorithm, which can improve precise individual therapy and prolong survival [6]. Her previous research also confirmed it is an effective and safe method in the evaluation of hypermetabolic bone lesions in patients with suspected advanced lung cancer [23]. Bing et al. drew a conclusion that PET/CT-guided targeted BMB may complement the results of possible false-positive PET/CT and false-negative iliac crest biopsy findings for evaluation of bone marrow involvement in newly diagnosed lymphomas [7].

However, there are fewer PET/CT apparatuses, thus their application is restricted. Furthermore, SPECT/CT is more common. Owing to that SPECT/CT is organic fusion of metabolic imaging and anatomical imaging, using SPECT/CT-guided biopsy can theoretically increase the accuracy and success rates and can be extensively promoted.There are fewer reports of SPECT/CT-guided biopsy. Zhao et al. applied SPECT/CT for thoracic tumour biopsy and confirmed its safety and reliability [8].

This study applied SPECT/CT to guide biopsy for suspected bone metastatic lesions in breast cancer. In our study, the relatively safer puncture site that is suspicious on SPECT/CT images was preferred consideration. Thus, the biopsy success rate was 100%, there were no serious complications, and an adequate amount of tissue was obtained in all 49 patients. 5 of 49 patients had no morphological changes on CT and were thus not suitable for CT-guided biopsy, while SPECT/CT identified an accurate biopsy site. Statistical analysis demonstrated that SPECT/CT-guided BMB showed significantly higher sensitivity and NPV when compared to SPECT/CT for determination of BM. Methods, results, advantages and potential limitations of reported previous studies to evaluate metastasis involvement in breast cancer and the present study are summarized in Table 4.

**Table 4:** Methods, results, advantages and potential limitations of reported previous studies to evaluate metastasis involvement in breast cancer and the present study

Reference First author, year	Methods	Positive samples for metastatic breast cancer	Samples with adequate number of tumor cells for receptor analysis	Potential limitations
J. F. Hilton, 2011 [14]	CT-guided bone biopsy	21/39(52.5%)	19/39(48.8%)	Cannot confirm the location for tumors at the early stage
Wei Guo,2018 [6]	PET/CT guided biopsy	46/54(85.2%)	23/46(50%)	More expensive and less equipment
This study	SPECT/CT guided bone biopsy	34/39(69%)	34/34(100%)	99mTc-MDP is not a specific tracer for metastatic disease

The possible reasons lie in the principle of 99mTc-MDP and the limitations of SPECT/CT. Abnormal accumulation of 99mTc-MDP is related to changes in local blood flow and osteoblastic activity, but does not reflect the true tumor burden in the bone marrow. The mechanism of accumulation means that the uptake of 99mTc-labeled diphosphonates is not specific for metastatic disease [24]. BS and SPECT/CT sometimes fail to distinguish BM from benign disease, including trauma, inflammation and primary tumor of bone [10].

The additional radiation dose in this study was from positioning CT. Because of the advantage of fusion images, the tube current and tube voltage were only 20 mA and 120 mV, respectively, and the effective radiation dose was approximately (1.9 ± 0.8) mSv, which was lower than the dose for one-time chest CT scans and doses in literature reports [25].

In our study, the changing rates between metastatic lesions and primary lesions for ER, PR, and HER-2 expression in 34 metastatic tumors were 32.3% (11/34), 47% (16/34), and 14.7% (5/39) respectively, and the changing rate in the molecular subtype was 54.3% (19/39), a finding that was basically consistent with that in literature reports [6, 26].

In summary, this study suggested that SPECT/CT-guided bone biopsy was safe and feasible and did not significantly increase the radiation dose. It provides breast cancer patients with an opportunity for accurate pathological and heterogeneous diagnosis of suspicious BM. It has high clinical value and is worthy of extensive clinical application.

The limitations of our study were inherent to its retrospective design. In addition, conventional decalcification in bone biopsy histopathology might influence immunohistochemical results, therefore, the ER, PR, and HER-2 expression results might not be accurate.

## Conclusion

It is insufficient to evaluate BM in BC using 99mTC-MDP SPECT/CT imaging. It is recommended that SPECT/CT-guided BMB be performed if positive lesions were identified on SPECT/CT imaging, which could offer significantly higher sensitivity and NPV when compared to SPECT/CT for detection of BM in BC patients. In addition, our study initially showed that SPECT/CT guidance provided a new integrated approach for bone metastatic BC patients, which include diagnosis of bone lesions, as well as a new SPECT/CT-guided BMB method to achieve tissue samples for monitoring potential molecular subtyping shifts of BC.

The integrated approach that includes SPECT/CT and SPECT/CT-guided BMB, which can be performed in one stop in nuclear medicine department, can offer important opportunities for precision treatment and improved quality of life of breast cancer patients with BM.

## Data Availability

All data generated or analysed during this study are included in this published article.

## Abbreviations

BC: Breast cancer
BM: Bone metastases
BMB: Bone marrow biopsy
NCCN: National Comprehensive Cancer Network
ESMO: European Society for Medical Oncology
CACA: China Anti-cancer Association
MDP: 99Tcm-methyl diphosphonate
BS: Bone scintigraphy
ER: Based on estrogen receptor
PR: Progesterone receptor
HER2: Human epidermal growth factor receptor 2
DLP: Dose-length product
PPV: Positive predictive value
NPV: Negative predictive value
